# Bioinspired Nanoemulsions Stabilized by Phosphoethanolamine and Phosphoglycerol Lipids

**DOI:** 10.3390/nano10061185

**Published:** 2020-06-18

**Authors:** Carlo Caianiello, Marcellino D’Avino, Domenico Cavasso, Luigi Paduano, Gerardino D’Errico

**Affiliations:** 1 Department of Chemical Sciences, University of Naples Federico II, Via Cintia 4, Complesso Universitario di Monte Sant’Angelo, I-80126 Naples, Italy; carl.caianiello@studenti.unina.it (C.C.); marce.davino@studenti.unina.it (M.D.); domenico.cavasso@unina.it (D.C.); luigi.paduano@unina.it (L.P.); 2 CSGI, Consorzio Interuniversitario per lo Sviluppo dei Sistemi a Grande Interfase, Via della Lastruccia 3, I-50019 Sesto Fiorentino, Florence, Italy

**Keywords:** nanoemulsion, phospholipid, dynamic light scattering, electron paramagnetic resonance

## Abstract

Water-in-oil (W/O) nanoemulsions stabilized by phospholipids (PLs) are increasingly exploited in a wide spectrum of applications, from pharmaceuticals to food and cosmetic formulations. In this work, we report the design and optimization of an innovative emulsion based on a mixture of phosphoethanolamine (PE) and phosphoglycerol (PG) PLs, inspired by the composition of the inner leaflet of a bacterial outer membrane. Using the natural oil squalene as the continuous organic phase, no additional emulsion stabilizer is needed. On the other hand, a small amount of Span 80 is required when dodecane is used. The obtained nanoemulsions are stable for at least two hours, thus allowing the droplet size and distribution to be characterized by Dynamic Light Scattering (DLS) and the lipid layer structure and dynamics to be analyzed by Electron Paramagnetic Resonance (EPR) spectroscopy. The results indicate that squalene shallowly intercalates among the lipid tail termini, being unable to deeply penetrate the adsorbed lipid monolayer. The altered lipid dynamics are proposed to be the reason for the enhanced emulsion stability, this paving the way to future implementations and possible applications.

## 1. Introduction

Phospholipids (PLs), one of the major structural elements of biological membranes, are unique natural surfactants. Their biocompatibility, along with outstanding interfacial properties, drives their application as emulsion stabilizers in food, pharmaceutical and cosmetic formulations [1].

As a matter of fact, direct oil-in-water (O/W) emulsions stabilized by phospholipids find a variety of applications in food production, drug delivery and medicine. Lecithins, natural mixtures with a predominant phosphocholine (PC) component, are by far the most used phospholipids in the food industry. They are largely used in chocolate production, where, combined with coemulsifiers, they act as rheology modifiers. Hydrolyzed lecithins are also included in caramel and chewing-gum formulations, where they help with ingredients’ homogenization during manufacture and prevent sugar recrystallization during shelf life of the finished products [2]. In the pharmaceutical field, PL-stabilized O/W emulsions are widely used for intravenous feeding and the delivery of fat-soluble anesthetic compounds [3].

Due to their wide use, O/W PLs emulsion have been extensively investigated. Their stabilization mechanism is a complex process affected by several factors such as the structure of the absorbed lipid monolayer at phase boundary, the lipid adsorption kinetics and intermolecular forces; it is indeed a broadly covered topic in the published literature [4,5,6,7]. Friberg et al. proved that emulsion stability is enhanced by the presence of a PLs liquid crystalline layer at the interface as well as by electrostatic repulsion. These authors also showed that increasing the repulsion between oil droplets or, on the other hand, reducing their attraction is more important in preserving emulsion long-term stability than the presence of single or multilayer at the interface [4]. O/W emulsion stability was also investigated as a function of the interfacial and bulk properties. PC as emulsifier proved to provide much longer stability than phosphoethanolamines (PE) and phosphoserines (PS) because it is able to form a more compact monolayer at the interface. Indeed, the monolayer formed by PC is in a liquid-condensed state while those formed by PE and PS are in a liquid-expanded phase. For this reason, a large number of oil molecules are present amongst the interfacial boundary when PE and PS are used as emulsifiers, thus favoring droplets’ coalescence [5]. Since O/W emulsion stabilized by PLs is extensively used as vehicle for drug delivery, several authors have studied lipid–drug interactions, thus showing how any perturbation of the absorbed interfacial structure affects the emulsification process outcome. In particular, Nordén et al. have shown that chlormethiazole, used as a model drug, preferentially sets close to the PLs layer and highly interacts with it, thus both depressing the PLs transition temperature and influencing the droplet size of the dispersed phase [6].

With respect to O/W emulsions, inverse water-in-oil (W/O) emulsions stabilized by PLs have been less investigated [7]. W/O emulsions generally show low stability since only steric repulsion plays a significative role in the stabilization process. Indeed, electrostatic repulsion among dispersed droplets is expected to be almost negligible because of the low electric conductivity of the organic continuous phase [8]. In principle, PLs could be expected to behave as inverse emulsion stabilizers due to (i) their geometrical shape, mostly comparable to a truncated cone or a cylinder, (ii) their high solubility in organic solvents and (iii) their hydrophilic-lipophilic balance (HLB) value, the latter usually being in the optimal range of a W/O emulsifier. Notwithstanding, PLs W/O emulsions are commonly reported to be unstable; in fact, only emulsions characterized by dilute dispersed phases (i.e., less than 1% of water [9]) show a certain stability, ranging from minutes to hours [10]. 

The reasons for W/O PLs emulsion instability have been investigated by Sommerling et al. [10]. These authors found an important effect of the critical aggregation concentration (CAC) both on the long-term stability and on the mechanism governing the emulsion temporal evolution (e.g., Ostwald ripening or coalescence). Above the CAC, PL reverse micelles form, which act as water carriers among the emulsion droplets. Thus, in order to achieve relatively stable droplets dimension lower than 500 nm, phospholipids’ concentration should be lower than their CAC. 

The abovementioned instability problems well deserve further efforts to be fixed. Indeed, W/O PLs emulsion could find important applications in the pharmaceutical field (as carriers aiming at enhanced bioavailability of drugs or contrast agents for biomedical imaging [11]) and in the functional food design (allowing the reduction of the fat content, the encapsulation of hydrophilic bioactive compounds and their controlled release [12]). Moreover, they have great potential as intermediates in the preparation of specifically engineered asymmetric vesicles, designed to be realistic models of bio-membranes [13]. For all these applications, nanoemulsions (i.e., submicrometer-sized emulsions with droplet size ranging 50–200 nm [14]) hold considerable potential. As a matter of fact, these colloidal systems exhibit high kinetic stability because of the small droplet size. Indeed, it appears that Brownian motion is able to overcome the gravity forces that drive the system separation through creaming and sedimentation. In addition to their high kinetic stability, another important feature is the wide surface area providing better drug bioavailability [15]. 

In this work, we show that a promising strategy to obtain more stable W/O nanoemulsions is the design of a system able to mimic a stable biological structure, i.e., a lipid layer with negative curvature. This approach is part of a current research trend, known as “bioinspiration” [16], where nature is used as a guiding line for the design of molecules or supramolecular aggregates. In particular, the inner leaflet of the Gram-negative bacteria’s outer membrane has an architecture suitable for our purpose. Gram-negative bacteria’s outer membrane (OM) shows a strong asymmetric structure, in which the two opposing leaflets differ in their lipid composition. The external leaflet is populated by lipopolysaccharides while the inner one is highly rich in phosphoethanolamines and phosphatidylglycerols [17]. Thus, the lipid composition of the inner leaflet has been evolutionarily optimized to assume a spontaneous negative curvature [18]. 

On this basis, we investigated how W/O nanoemulsions stabilized a PLs mixture formed by 1-palmitoyl-2-oleoyl-sn-glycero-3-phosphoethanolamine (POPE)/1-palmitoyl-2-oleoyl-sn-glycero-3-phospho-(1’-rac-glycerol) (POPG) at 70/30 mol/mol; that is roughly the same composition of the inner leaflet of gram-negative bacteria’s OM. The effect on the emulsion’s stability of two continuous phases, i.e., dodecane and squalene, was evaluated. We monitored droplets dimension over time using dynamic light scattering (DLS) measurements, and PLs structuring at the droplet interface by means of electron paramagnetic resonance (EPR) spectroscopy using nitroxide-labelled PLs as molecular probes. Furthermore, we evaluated the impact of the inclusion of a small amount of a glycosylated surfactant, namely the Span 80, on the nanoemulsion stability.

Our efforts aim to get a better understanding of the interfacial structure at the droplet boundary as well as information on droplet dimensions and stability over time, thus contributing to build a robust scientific platform for possible applications of bioinspired PLs nanoemulsions.

## 2. Materials and Methods

### 2.1. Materials

**Figure 1:**
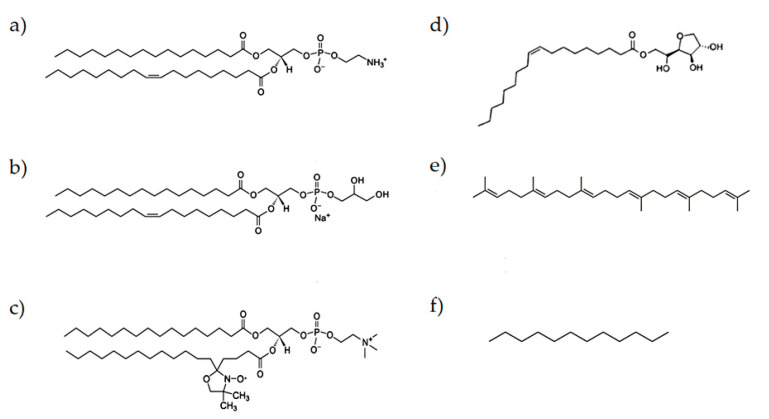
Molecular formulae of (**a**) POPE, (**b**) POPG, (**c**) 5-PCSL (chosen as an example of spin label) (**d**) Span 80, (**e**) squalene and (**f**) dodecane.

The molecular formulae of some substances used in this study are presented in Figure 1. Phospholipids 1-palmitoyl-2-oleoyl-sn-glycero-3-phosphoethanolamine (POPE) (>99% purity) and 1-palmitoyl-2-oleoyl-sn-glycero-3-phospho-(1′-rac-glycerol) (POPG) (>99% purity) were obtained from Avanti Polar (Birmingham, AL, USA). Sorbitane monooleate (Span 80) and dodecane (>99% purity) were purchased from Sigma–Aldrich (St. Louis, MO, USA). Squalene (98% purity) was purchased form Alfa Aesar (Ward Hill, MA, USA). Spin-labelled phosphatidylcholines (1-palmitoyl-2-stearoyl-(n-doxyl)-sn-glycero-3-phosphocholine, n-PCSL, n = 5, 7, 10, 14) (>99% purity), used for EPR measurements, were also purchased from Avanti Polar (Birmingham, AL, USA) and stored at −20 °C in ethanol solutions. Products were used without further purification. All aqueous solutions were prepared by using Millipore water.

### 2.2. Samples Preparation

For DLS and EPR measurements, emulsions with two different compositions were alternatively tested: POPE-POPG (70/30 mol/mol) and POPE-POPG-Span 80 where the ratio between POPE and POPG was fixed at 70/30 mol/mol while Span 80 was 5% of the overall lipid moles. Stock solutions were prepared by dissolving the right amount of phospholipids and Span 80 in chloroform. To obtain W/O emulsions a lipid suspension was preliminarily prepared. To prepare the lipid suspension, 43 μL of a 3 mg mL^−1^ POPE stock solution, 60 μL of a 1 mg mL^−1^ POPG stock solution and 2 μL of a 5 mg mL^−1^ Span 80 stock solution (where required) in chloroform were placed in a 20 mL glass vial. The chloroform was evaporated under nitrogen to obtain a dry, thin lipid film. After adding 4 mL of oil phase (dodecane or squalene) to reach a final overall lipid concentration of 0.05 mg mL^−1^, the suspension was sonicated in a sonic bath for 45 min and left overnight at 25 °C to ensure that the lipid molecules were completely dispersed in oil. The emulsion was prepared by adding 20 μL of water, that is, 0.5% vol/vol, to the lipid suspension and gently stirring the mixture with a magnetic stir bar for 1 h. Finally, the emulsion was sonicated using a Sonics Vibracell VCX 130 PB ultrasonic processor (Sonics&Materials, Newtown, CT, USA) equipped with a 3 mm titanium probe running at an amplitude of 40% for 20 min in an ice bath.

### 2.3. DLS Measurements

Droplets’ dimension distribution and emulsion stability were checked by DLS. In order to perform the DLS analysis, 200 μL of the emulsion sample were placed in a cuvette and, due to the sample opalescence, diluted with the continuous phase (dodecane or squalene) to 1 mL, that is, 1:5 dilution.

DLS measurements were performed with a homemade instrument composed by a compact goniometer (Photocor Ltd., Moscow, Russia), a SMD 6000 50 mW light source (Laser Quantum, Fremont, CA, USA) operating at 5325 Å, a photomultiplier (PMT-120-OP/B) and a correlator (Flex02-01D) (Correlator.com, China). All the experiments were performed at (25.00 ± 0.05) °C, at a scattering angle θ = 90°. The scattered intensity correlation function was analyzed using a regularization algorithm [19,20]. The diffusion coefficient of each population of diffusing particles was calculated as the z-average of the diffusion coefficients of the corresponding distributions [21]. Considering that the mixtures are diluted, the Stokes–Einstein equation was used to evaluate the hydrodynamic radius, *R_H_*, of the aggregates from their translational diffusion coefficient, *D*:

(1) $$R_{H}=\frac{T\;k_{B}}{6\pi \;\eta_{0}\;D}\;$$

where *k_B_* is the Boltzmann constant, *T* is the absolute temperature, and *η*_0_ is the medium viscosity whose value was assumed to be that of the neat organic solvent (1.343 mPa·s for dodecane [22] and 12 mPa·s for squalene [23]).

### 2.4. EPR Characterization

EPR measurements were carried out to study the structuring and micropolarity of the lipid layer, by using spin-labelled phosphatidylcholines as probes. Although the conventional procedure establishes that the probing agent has to be incorporated in the phospholipid mixture before its drying for the thin film preparation [24], we adopted a new method where the probe was added a posteriori, after the emulsion has been prepared. Indeed, we observed that, when using the conventional procedure, the probe was only partially incorporated in the investigated system, leading to EPR spectra characterized by the superposition of two different components where the most predominant one was purely isotropic. This suggests that most of the recorded signal arose from radicals rapidly and randomly tumbling in the solvent. On the other hand, this adding of the probe a posteriori procedure led to anisotropic EPR spectra typical of nitroxide radical embedded in an ordered structure (i.e., the lipid layer at the w/o interface). Accordingly, emulsion samples for EPR analysis were prepared as follows: a proper amount of the previously prepared emulsion, right after the sonication step, was added to a thin probe film to reach a probe concentration of 1% by mass with respect to the lipids and eventually mixed using a vortex (ArgoLab Mix, Carpi, Italy). The probe thin film had been previously obtained by drying under nitrogen 2 μL of a 1 mg mL^−1^ placed in a 4 mL glass vial. The same procedure was repeated for every chosen spin-labelled phosphatidylcholine. The obtained probe-containing emulsion was finally transferred to a 25 μL glass capillary and flame sealed.

EPR analyses were executed both on the W/O emulsion and on water-suspended vesicles of the same lipid composition, where the latter were obtained by hydration of a phospholipids thin film and taken as reference. In the case of the vesicles, the spin-labelled lipid had been added directly to the initial POPE-POPG-Span 80 film to a concentration of 1% by mass with respect to the lipids.

ESR spectra were recorded with a 9 GHz Bruker Elexys E-500 spectrometer (Bruker, Rheinstetten, Germany). The capillaries were placed in a standard 4 mm quartz sample tube containing light silicone oil for thermal stability. All the measurements were performed at 25.0 ± 0.1 °C. Spectra were recorded using the following instrumental settings: sweep width, 120 G; resolution, 1024 points; time constant, 20.48 ms; modulation frequency, 100 kHz; modulation amplitude, 1.0 G; incident power, 6.37 mW. Several scans, typically 128, were accumulated to improve the signal-to-noise ratio.

### 2.5. Interfacial Tension Measurements

Interfacial tension measurements have been carried out in order to verify if the recorded values for our investigated systems were low enough to provide spontaneous emulsification. Water–oil interfacial tension has been recorded in the presence and absence of phospholipids. Phospholipids were added to the oil phase where their concentration was fixed at 0.05 mg mL^−1^ to simulate the same emulsion conditions. The oil (4 mL) was then gently brought to contact with the aqueous phase (10 mL), waiting for 24 h to allow the neat stratification of the organic phase over the aqueous medium and the formation of a lipid monolayer at the interface. Interfacial tension measurements were recorded with a Sigma 70 tensiometer (KSV Instruments LTD, Finland) equipped with the Du Nouy ring. Based on the standard method for surface tension measurement which involves the slowly lifting a platinum ring from the surface of a liquid, we used a procedure suitable for interfacial tension measurement. Specifically, the ring was gently submerged in the bottom heavier phase and then slowly lifted through the phase boundary. The force required to raise the ring from the liquid interface was measured and related to the interfacial tension. 

### 2.6. Statistical Analysis

DLS, EPR and interfacial tension measurements were repeated at least thrice on independently-prepared samples with the same nominal composition. The results are reported as mean value ± standard deviation.

## 3. Results and Discussion

### 3.1. Emulsion Design

The formulation of W/O inverted emulsions involved the optimization of several variables such as: the PLs mixture, the organic continuous phase, the PLs’ concentration, and the amount of water.

Among the frequently used PLs, such as PCs, which cannot act as emulsifiers themselves [25,26], the chosen PLs mixture (POPE-POPG at 70/30 mol/mol), besides being in agreement with our bioinspiration purpose, should potentially behave properly as an emulsion stabilizer. As a matter of fact, this mixture exhibits specific physico-chemical properties suitable for this aim, such as: oil-solubility, low HLB value and phase behavior. Indeed, both lipids are highly soluble in organic solvents (e.g., chloroform and methanol), but slightly soluble in water. PEs are reported to have HLB values around 6, while PGs’ HLB values are even lower [27,28]; hence their mixtures are expected to have HLB values which would promote their use as inverted emulsion stabilizers. Moreover, the PLs mixture employed is characterized by a thermotropic phase transition of the lipid microscopic arrangement from a gel phase to a fluid liquid crystalline phase at 18 °C [29]. Therefore, at 25 °C (i.e., our experimental temperature), PLs are in a fluid liquid crystalline phase thus potentially providing a major stability by wrapping the dispersed emulsion droplets in a viscous liquid crystalline matrix [30].

The choice of the organic phase, namely the dispersion medium of the inverted emulsion, is extremely important and is governed by the balance between the solubility of the lipid and the stability of the inverted emulsion. Generally, alkanes with chains shorter than 10 carbons lead to the formation of highly polydisperse microemulsions where it is not possible to control with reasonable accuracy the droplet size [9]. Taking this into account, we decided to use dodecane. Aiming at a more bioinspired choice of the organic phase, we also tested squalene, a natural compound which is extracted either from shark liver oil or vegetable oil. Squalene, a 30-carbon atom triterpene, is a highly viscous liquid which has been reported to stabilize W/O emulsion [9,10,11,12,13,14,15,16,17,18,19,20,21,22,23,24,25,26,27,28,29,30,31].

The choice of the amount of water (i.e., the dispersed phase) is also crucial since it significantly affects emulsion stability. At high water contents (i.e., for volume fraction higher than 1%) phospholipids can partition between water and oil [9]. The heavier water phase, consisting of big emulsion droplets, would quickly set at the bottom. Consequently, the droplets would coalesce, thus leading to fast emulsion destabilization. Therefore, in order to avoid a rapid coalescence, our emulsions contained no more than 0.5 vol % of water.

PLs concentration influences both emulsion stability and droplets size. It has been proved that at low PLs concentration (i.e., lower than their CAC), PLs are expected to stabilize smaller water droplets [10]. Therefore, to provide stable nanoemulsions in our experiments PLs concentration was always fixed at 0.05 mg mL^−1^, that is below our PLs CAC. Indeed, for PG and PE the CAC is generally in the micromolar range [32,33]. In our systems, the amount of PLs was in the order of a few nanomoles per liter.

As a last step in the emulsion design process, interfacial tension measurements have been carried out in order to optimize the preparation protocol. The obtained γ values are shown in Table 1. Spontaneous emulsification (i.e., thermodynamic stability) occurrs only when interfacial tension is as low as 0.01 mN m^−1^ [34]. Inspection of the table shows that, even though the lipid mixtures are effective in lowering the γ values for both the considered oil/water pairs, still the values are three orders of magnitude higher than 0.01 mN m^−1^. Thus, the investigated nanoemulsion can be only kinetically stable and a significant energy supply is needed for the emulsification process. For this reason, we decided to use a sonication by a tip probe for quite a long time (20 min) in an ice bath.

### 3.2. Dynamic Light Scattering

**Figure 2:**
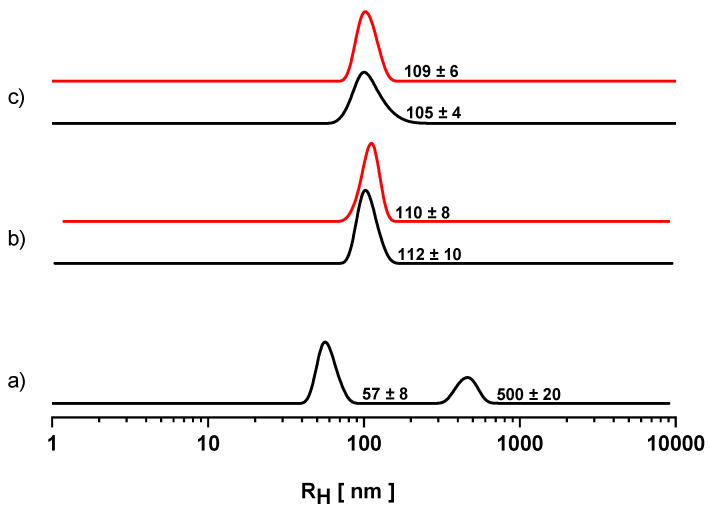
Hydrodynamic radius distributions for water-in-oil (W/O) emulsions measured at time zero (t_0_), that is, immediately after emulsification (black lines), and one hour later (red lines). (**a**) Water/dodecane POPE-POPG emulsions; (**b**) water/dodecane POPE-POPG-Span80 emulsions; (**c**) water/squalene POPE-POPG emulsions.

Water/dodecane inverted emulsions stabilized by POPE-POPG have been initially investigated. Droplet dimension distribution was evaluated by DLS immediately after sonication and over time. DLS provides information about the scatterers’ motion in terms of diffusion coefficient, from which it is possible to calculate the hydrodynamic radius by applying the Stokes–Einstein equation Equation (1). The results are reported in Figure 2a where the scattering intensity distribution has been normalized by assuming a dependency of the mass (*M*) of the droplets with respect to their hydrodynamic radius as *M* ∝ *R_H_*^3^. As can be seen, a bimodal distribution is observed, with one population being significantly smaller than the other. The dispersed phase is mostly distributed into small droplets, whose radii is lower than 100 nm. This observation is in agreement with recent findings showing that, when the concentration of PLs is far lower than their CAC, they form a single monolayer at the interface giving rise to smaller droplets less prone to coalescence during emulsification [10]. However, the presence of a second population of larger droplets clearly points to an unsatisfactory emulsification. Indeed, the water/dodecane POPE-POPG emulsion exhibited a very low stability: a macroscopic phase separation occurred in a few minutes, most likely driven by the fast coalescence of the bigger emulsion droplets. This does not allow the measurements to be carried out over time.

Facing this instability problem, we tested two alternative approaches. The first strategy consists in optimizing the lipid mixture by adding specific stabilizing components. The second one consists in changing the oil-phase.

By following the first approach, we redesigned the emulsifier system by adding a small amount of a glycosylated surfactant to the original lipid mixture with the aim to provide a more viscous external coating for the water droplets, thus preventing their coalescence. We chose Span 80, one of the most diffuse W/O emulsifiers. 5% (mol/mol) of Span 80 was added to the lipid mixture during the film preparation while the lipid concentration was still fixed at 0.05 mg mL^−1^. Once formulated, the water/dodecane POPE-POPG-Span 80 emulsion was then characterized. The droplet size distribution was determined by DLS. The hydrodynamic radii distribution was measured immediately after sonication (t_0_) and over time (three times a step of 30 min). The results at t_0_ and after 1 h are shown in Figure 2b. The system is characterized by a single monomodal distribution hinged at about 110 nm (black line). The observed population is unchanged after 1 h (red line). The same result was obtained even after 90 min (data not shown). Indeed, there was no clear phase separation for at least two hours. Thus, Span 80 greatly improves emulsion droplet dispersion as well as stability. This result has to be ascribed to the specific Span 80 features. Its low HLB value of 4.3 [35] along with its high solubility in organic solvents makes it suitable for W/O inverted emulsion stabilization. Presenting a relatively small size, it rapidly diffuses at the interface creating a compact superficial coating. The long hydrocarbon chain stretches out from the absorbed interfacial layer, pointing towards the continuous phase, and creates a strong steric barrier. In this way, as two droplets approach each other, coalescence is hindered by the unfavored interpenetration of the lipid layers, thereby resulting in entropic repulsion [36].

Even though this first strategy proved to be effective in enhancing emulsion stability, Span 80 inevitably changes the absorbed lipidic layer composition at water droplets boundary thus compromising the declared bioinspiration purpose. As an alternative approach, we changed the dispersion medium substituting squalene to dodecane. In this case the emulsions were formulated without adding any stabilizers like Span 80. Measurements have been carried out with the same frequency as before and the effects on the emulsification process (i.e., droplets’ size and emulsion stability) of squalene as a continuous phase are presented in Figure 2c. One monomodal distribution is recorded at t_0_, namely immediately after sonication (black line) centered at 100 nm. This population is still observed after one hour (red line) and also after 90 min from t_0_ (data not shown); even in this case, no phase separation was detected for at least two hours. It is clear that the different continuous phase has strongly influenced the emulsification results. The increased stability has to be ascribed to the higher viscosity of the squalene (12 cP) [23] compared to the dodecane one (1.34 cP) [22]. The water droplets’ diffusion in the dispersion medium is lowered by the increased viscosity of the continuous phase, as described by equation 1. A low diffusion coefficient reduces the number of collisions, so the rate of coalescence becomes smaller [9], thus leading to major stability over time.

Overall, both adopted strategies led to the formation of stable W/O emulsion whose droplets size distribution was fixed in the range of 100 nm. In other terms, stable W/O Pls nanoemulsions have been successfully formulated.

### 3.3. Electron Paramagnetic Resonance

The addition of Span 80 to the original POPE-POPG lipid matrix as well as the substitution of the continuous phase (squalene in lieu of dodecane) made W/O PLs emulsions stable enough to be characterized by means of EPR spectroscopy.

EPR measurements were carried out to analyze the microstructure of the lipid layer adsorbed at the boundary of the water droplets, by incorporating in it phosphatidylcholines spin-labeled at the different positions of the sn-2 chain (n-PCSL, with n = 5, 7, 10, 14). EPR analysis was performed both on the emulsions and on vesicles of the same lipid composition, eventually including Span 80. PLs vesicles, prepared with a well-established method (see Section 2.4), were chosen as a reference system. Indeed, while EPR is a well-consolidated approach to characterize lipid bilayers [37], it has been only seldom used for PLs reverse micelles [38]. To the best of our knowledge this is the first time that the same method is used for lipid monolayers stabilizing emulsion droplets. Each probe provides different information: 5-PCSL monitors the region just underneath the hydrophilic interface of the lipid layer (or bilayer), while in the case of 14-PCSL the reporter group is either directed towards the oil phase or deeply embedded in the membrane hydrophobic core, in the case of emulsions or lipid bilayers, respectively [39].

**Figure 3:**
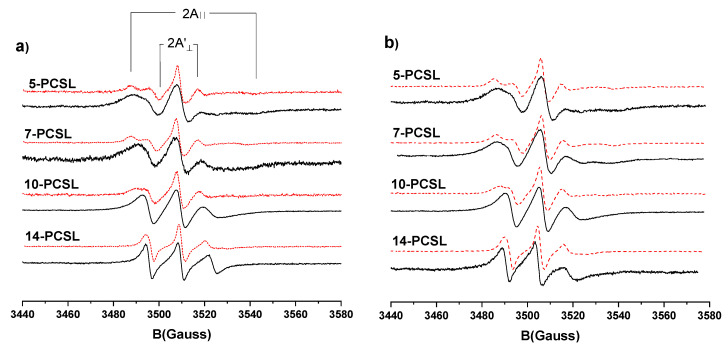
(**a**) Electron paramagnetic resonance (EPR) spectra of n-PCSL in POPE-POPG-Span 80 water/dodecane emulsions (solid black line) and in POPE-POPG-Span 80 vesicles (dotted red line). (**b**) EPR spectra of n-PCSL in POPE-POPG water/squalene emulsion (solid black line) and in POPE-POPG (dotted red line) vesicles.

First, n-PCSL spectra in POPE-POPG-Span 80 water/dodecane emulsion and in POPE-POPG-Span 80 vesicles were recorded, see Figure 3a. For all the labels, no evidence of superimposed spectra arising for the label partitioning between different environments is observed, thus confirming the labelled lipids reside exclusively at the W/O interface, participating to the Pls adsorbed monolayer.

Focusing our attention on the results obtained for the water/dodecane emulsion (black solid lines in Figure 3a) we note that the 5-PCSL spectrum shows an evident anisotropic lineshape. The low field signal is split in two peaks, which, however, are partially overlapped, while at the high field region, two separate minima can be clearly identified. In the case of 7-PCSL the two peaks at low field coalesce in one maximum with a shoulder on the left-hand-side, while in the high field region an unresolved extremely broad signal is detected. The spectra of 10 and 14-PCSL show only three broad signals typical of a slow, isotropic motion of the label. The lines become narrower, shifting from 10 to 14-PCSL. In summary, the spectra of n-PCSL embedded in the POPE-POPG-Span80 monolayer adsorbed at the water/dodecane interface of the inverted emulsion exhibit a gradient of the anisotropy of the label motion in going from the lipid headgroups to the tail termini. This reflects an increasing rotational freedom of the acyl chain segments when moving from the constrained water/oil interface to the continuous apolar environment.

It is interesting to compare these spectra with those recorded for n-PCSL spin-labels embedded in POPE-POPG-Span 80 vesicles (dotted red lines in Figure 3a). In this case, the 5-PCSL spectrum shows a marked anisotropic lineshape. The signal splitting both in the high and low field region is clearly resolved. The anisotropy is preserved in the 7-PCSL spectrum and still well distinguishable for 10-PCSL. In the 14-PCSL spectrum, the two peaks at low field are no longer resolved, thus coalescing in a single maximum, while at high field region a partial anisotropy is still detectable. Overall, the n-PCSL spectra observed in the bilayer show an evident mobility gradient in going from the membrane interface to the inner core. The comparison of the spectra shows that the acyl chains of the PLs absorbed at the boundary of the emulsion water droplets, with respect to the same molecules organized in bilayers, are far more capable of rotational motion. Indeed, the anisotropy is less marked at all label positions. This indicates that they are involved in a less tightly self-organized structure. Further details can be achieved by a quantitative analysis of the spectra. A parametrization of the n-PCSL spectra was realized determining the order parameter, *S*, and the hyperfine coupling constant, *a’_N_*. These parameters can be evaluated from the distance (in G) between maxima and minima of the spectra (see Figure 3a) using the following equations:

(2) $$S=\frac{A_{∥}-A_{⊥}}{A_{\mathrm{zz}}-A_{\mathrm{xx}}}\frac{A_{0}}{A_{0}^{\prime }}$$

(3) $${a^{\prime }}_{N}=\frac{1}{3}\left(A_{∥}+2A_{⊥}\right)$$

**Figure 4:**
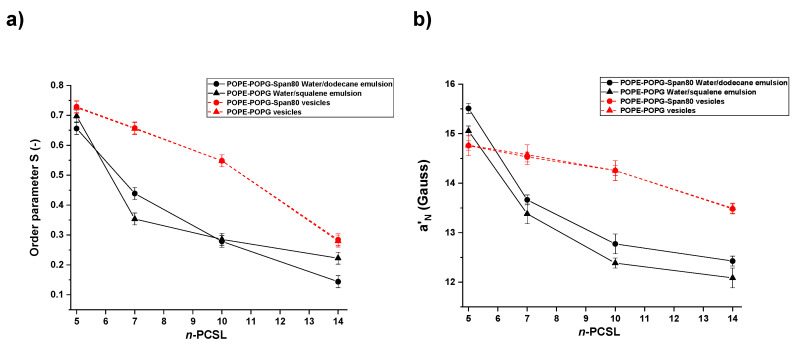
Dependence on spin-label position, n, of the order parameter, S (**a**), and of the micropolarity index *a’_N_* (**b**), of the n-PCSL phosphatidylcholine spin labels in: POPE-POPG vesicles (▲, dotted red line); POPE-POPG-Span 80 vesicles (●, dotted red line); POPE-POPG water/squalene emulsion (▲, solid black line); POPE-POPG-Span 80 water/dodecane emulsion (●, solid black line).

*S*, which express the angular amplitude of the motion, is a measure of the local orientational ordering of the labeled segment of the lipid tail with respect to the normal to the absorbed monolayer/bilayer surface, while *a’_N_* is an index of the micropolarity experienced by the nitroxide radical [40]. Figure 4a,b show the dependence of *S* and *a’_N_* on the label position along the chain, n, for n-PCSL in POPE-POPG-Span 80 water/dodecane emulsion (black circles, solid black line) and POPE-POPG-Span 80 vesicles (red circle, dotted red line). Inspection of the figure reveals an *S* decreasing trend for both the emulsion and the vesicle suspension. The *S* values obtained for the adsorbed monolayer are always lower than those relative to the lipid bilayer. This quantitatively demonstrates a higher acyl chain mobility due to a looser structuring of the lipids in the former case. It is interesting to observe that in the adsorbed monolayer the *S* decrease is steeper close to the lipid headgroups while in the bilayer the largest S drop between 10-PCSL and 14-PCSL. Indeed, the lipid packing of phospholipid chains in a bilayer produces a strong reduction of their mobility, while the adsorbed monolayer presents a much more disordered organization. This result clearly highlights the strict interconnection between the lipid organization, at the microscopic level, and the aggregate morphology, at the mesoscopic level.

The *a’_N_* trends also show a decrease for both emulsion and vesicles. This is consistent with the *a’_N_* physical meaning, this parameter being related to the micropolarity of the local environment in which the reporter group is embedded. Indeed, it can be seen that *a’_N_* decreases as the nitroxide group is shifted along the chain, corresponding to a deeper localization either in the oil continuous phase or in the hydrophobic interior of the phospholipid bilayer. However, it is interesting to observe that the overall *a’_N_* variation is much larger in the emulsion than in the membrane. Indeed, for 10- and 14-PCSL, the *a’_N_* are very low, indicating that the microenvironment of the lipid monolayer stabilizing emulsion droplets is much more hydrophobic than the interior of the lipid bilayers. At the same time, the polarity gradient across the lipid layer is much steeper in the emulsion than in the membrane, particularly close to the lipids headgroups. This difference has to be connected to the different lipid self-organization. Because of the great conformational freedom of the PLs acyl chains, the hydrophobic section of the adsorbed monolayer resembles an apolar liquid almost impermeable to water molecules. Moreover, the dodecane molecules can establish hydrophobic interaction with the phospholipid acyl chains, thus being able to solubilize in the Pls monolayer. This leads to a much more hydrophobic environment experienced by the nitroxide labels in the layer interior and, at the same time, to a disordered tail packing. Interestingly, the higher disorder favors the penetration of water molecules close to the headgroup region, as highlighted by the higher *a’_N_* observed for 5-PCSL.

n-PCSL spectra in POPE-POPG water/squalene emulsion and in POPE-POPG vesicles were also recorded—see Figure 3b. Inspection of the figure shows that substituting the dispersion medium (squalene in lieu of dodecane) as well as changing the emulsifier mixture composition (removing Span 80) does not cause dramatic changes in n-PCSL spectra with respect to the previously analyzed system. The only noteworthy difference is a significant variation of the 14-PCSL spectrum in the emulsion. Indeed, while the two peaks at low field are still not resolved, in the high field region a first peak is followed by a broad, partially unresolved, signal. This indicates that for 14-PCSL in POPE-POPG water/squalene emulsion a partial anisotropy is still preserved, thus suggesting a more restricted motion of the phospholipids chain termini. This restriction likely derives from a partial intercalation of the bulky squalene molecules among the tails. A similar evidence is not detectable in the 7-PCSL spectrum, thus indicating the squalene entering in the monolayer to be limited to its oil-exposed boundary. On the other hand, for n-PCSL spin-labels incorporated in POPE-POPG vesicles (dotted red lines in Figure 3b), no difference has been observed with respect to the POPE-POPG-Span 80 vesicles, the spectra being perfectly superimposable. This implies that inclusion of Span 80 does not interfere or perturb the lipid microscopic organization of the bilayer.

Even for this system, the spectra were quantitatively analyzed by the parametrization approach. The dependence of *S* and *a*’*_N_* on the label position along the chain for the n-PCSL spin-labels in POPE-POPG water/squalene emulsion (black triangle, solid black line) and POPE-POPG-Span 80 vesicles (red triangles, dotted red lines) is reported in Figure 4a,b. Analysis of the figures leads to conclusions qualitatively similar to those reached for the previously analyzed system. Indeed, even for POPE-POPG water/squalene emulsion the *S* values show a steep decreasing trend while *a*’*_N_* exhibits a larger gradient across the lipid layer than in the membrane. However, significant differences arise by quantitatively comparing the *S* and *a*’*_N_* values.

The *S* trends are slightly different. In the presence of squalene as dispersion medium, moving the probing group down along the acyl chains, *S* immediately decreases but then tends to stabilize, maintaining in the case of 14-PCSL a higher value than observed using dodecane as the oil. This is most likely caused by the insertion of the bulky and sterically hindered squalene molecules among the lipid tail termini, which leads to a decrease in the chain termini mobility [41]. However, because of its molecular structure, squalene is not able to be enter into the monolayer interior. The latter consideration is corroborated by literature data showing that squalene allows lipid self-aggregates to be oil-free [9]. Even in the lack of oil penetration, the monolayer interior is extremely hydrophobic. Indeed, the *a*’*_N_* values obtained for the POPE-POPG water/squalene emulsion are lower than those relative to the POPE-POPG-Span 80 water/dodecane emulsion. This evidence could be ascribed to the disordered organization of the lipids (confirmed by the low S values observed for 7-PCSL), forming a liquid-like apolar environment.

It has to be remarked that the values of *S* and *a*’*_N_* of the membranes in the presence and absence of Span 80 are perfectly matching, thus giving a quantitative confirmation of Span 80 incorporation without disturbing the PLs’ local structure.

## 4. Conclusions

Emulsions are hierarchically structured systems: their macroscopic stability depends on the mesoscopic dimension and the distribution of the dispersed droplets, which are affected by the microscopic structure and composition of the droplet–continuous medium interface, which in turn are determined by the molecular features of the emulsifiers [42].

In this work, we focused on the microscopic organization of the emulsifiers POPE and POPG in PLs-stabilized W/O nanoemulsions. These lipids were chosen because they are the components of the inner leaflet of the outer membrane of Gram-negative bacteria, a biological structure with a spontaneous negative curvature. We demonstrated EPR spectroscopy, by using proper spin-labelled phospholipids, to be suitable approach to deeply analyze the structure and dynamics of the lipid monolayers adsorbed at the droplet interface. POPE and POPG are positioned with the headgroups towards the aqueous pool, while tails form a poorly structured hydrophobic layer surrounding the droplet. Similar results were obtained using two different oil phases, dodecane and squalene. The latter oil, however, deserved more attention. Squalene has been reported to increase the stability of PLs W/O emulsion. Indeed, even in this work we found that, while an emulsion stabilizer (Span 80) is necessary to obtain relatively stable PLs water/dodecane nanoemulsions, no additional component is required when squalene is used as the oil phase. A deeper analysis of the EPR results allows some light to be shed on this point. Indeed, oil solubilization in the lipid layer was reported to be among the main destabilization mechanisms of PLs emulsions. The EPR results clearly show that the natural oil squalene is unable to deeply enter the lipid structure. Moreover, its shallow interaction among the acyl tail termini locally reduces the lipid dynamics. This results in a lipid monolayer in which the dynamics of both interfaces (the hydrophilic one formed by the headgroups and the hydrophobic one formed by the tail termini) is relatively hindered, while the monolayer core presents a highly disordered structure impermeable to both water and oil molecules. The oil-induced strengthening could underlie the enhanced stability of the nanoemulsions.

Overall, this study highlights that a bioinspired design is a suitable as a strategy to obtain more stable nanoemulsions and for general nanostructured formulations, thus fostering their profitable applications.

## Figures and Tables

**Table 1:** Water/oil interfacial tension values. The oil is either dodecane or squalene; the effect of the lipids POPE and POPG at 70:30 mol/mol is also considered.

System	Interfacial Tension γ (mN m^−1^)
dodecane/water	52 ± 1
squalene/water	49 ± 1
dodecane/water POPE-POPG	20 ± 1
squalene/water POPE-POPG	12 ± 1
